# Korean Red Ginseng Pretreatment Protects Against Long-Term Sensorimotor Deficits After Ischemic Stroke Likely Through Nrf2

**DOI:** 10.3389/fncel.2018.00074

**Published:** 2018-03-23

**Authors:** Lei Liu, Mary K. Vollmer, Victoria M. Fernandez, Yasmin Dweik, Hocheol Kim, Sylvain Doré

**Affiliations:** ^1^Department of Anesthesiology, Center for Translational Research in Neurodegenerative Disease and McKnight Brain Institute, University of Florida, Gainesville, FL, United States; ^2^Department of Herbal Pharmacology, College of Korean Medicine, Kyung Hee University, Seoul, South Korea; ^3^Departments of Neurology, Psychiatry, Pharmaceutics, Psychology and Neuroscience, University of Florida, Gainesville, FL, United States

**Keywords:** aquaporin 4, astrogliosis, neurological deficit, neuroprotection, oxidative stress, prevention, transcriptional factor Nrf2, recovery

## Abstract

Endogenous neuroprotective mechanisms by which the brain protects itself against noxious stimuli and recovers from ischemic damage are key targets of stroke research, ultimately facilitating functional recovery. Transcriptional factor Nrf2, enriched in astrocytes, is a master regulator of endogenous defense systems against oxidative stress and inflammation. Korean Red Ginseng (Ginseng), one most widely used herbal medicine, has exhibited promising potentials in neuroprotection. Our study aimed to determine whether the standardized Ginseng extract pretreatment could attenuate acute sensorimotor deficits and improve long-term functional recovery after ischemic stroke though Nrf2 pathway and whether reactive astrogliosis is associated with such effect. Adult Nrf2^−/−^ and matched wildtype control (WT) mice were pretreated with Ginseng orally for 7 days prior to permanent distal middle cerebral artery occlusion (pdMCAO). Using an optimized method that can accurately assess either severe or mild pdMCAO-induced sensorimotor deficits, neurobehavioral tests were performed over 28 days. The progression of lesion volume and the evolution of astrocytic and microglial activation were determined in the acute stage of ischemic stroke after pdMCAO (0–3 days). Nrf2-downstream target antioxidant genes expression levels was assessed by Western blot. We found that Ginseng pretreatment ameliorated acute sensorimotor deficits and promoted long-term functional recovery, prevented the acute enlargement of lesion volume (36.37 ± 7.45% on day 3), attenuated reactive astroglial progression but not microglia activation, and enhanced the induction of Nrf2-downstream target proteins after ischemic insult in WT mice, an effect which was lost in Nrf2 knockouts. The spatiotemporal pattern of reactive astrogliosis evaluation correlated well with acute ischemic damage progression in an Nrf2-dependent fashion during the acute phase of ischemia. In contrast, Nrf2 deficiency mice exhibited exacerbated ischemic condition compared to WT controls. Together, Ginseng pretreatment protects against acute sensorimotor deficits and promotes its long-term recovery after pdMCAO, at least partly, through Nrf2 activation, highlighting the potential efficacy of oral consumption of Ginseng for stroke preventative intervention in patients who are at great risk of recurrent stroke or transient ischemic attack. The attenuated reactive astrogliosis contributes to the Nrf2 pathway related neuroprotection against acute ischemic outcome and substantially long-term sensorimotor deficits in the context of ischemic stroke under pdMCAO.

## Introduction

Ischemic stroke, more often disabling than fatal, is the leading cause of severe and long-term disability in the United States (Mozaffarian et al., 2016). Endogenous neuroprotective mechanisms by which the brain protects itself against noxious stimuli and recovers from ischemic damage are key targets of stroke research, ultimately facilitating functional recovery. The transcriptional factor Nrf2 is a key regulator of endogenous defense system against oxidative stress and inflammation by upregulating cytoprotective genes encoding for phase II defense enzymes and antioxidant-related proteins, which are particularly enriched in astrocytes (Leonardo and Doré, 2011; Ma, 2013; Kumar et al., 2014). Because astrocytes have diverse and crucial functions in many aspects of ischemic brain damage, the activation of Nrf2 in astrocytes may confer neuronal protection by efficient antioxidant defense in ischemic stroke pathogenesis and the process of recovery. Such activation of antioxidant pathways is particularly important for neuroprotection in brain with relatively weak endogenous antioxidant defenses (Albarracin et al., 2012). Therefore, targeting Nrf2 has emerged as a promising therapeutic strategy for brain diseases prevention or reversal. Our group has been interested in screening various natural Nrf2 inducers for beneficial processes against ischemic stroke and other neurological disorders.

Korean Red Ginseng (Ginseng) is derived from the peeled and dried root of Panax ginseng, and its major active components are ginsenosides (Christensen, 2009; Kim et al., 2013). Ginseng has exhibited an encouraging protective efficacy against various neurological disorders in both preclinical and clinical studies. Although many of its purported effects have pointed to Ginseng as a potential preventive medicine with direct antioxidant and anti-inflammatory properties, the underlying cellular mechanisms are still unclear (Liu, 2012; Kim et al., 2013; Lee and Kim, 2014; Rastogi et al., 2014). Considering that the Nrf2 pathway also plays a vital role in oxidative stress and inflammation, we propose that Ginseng could act as a protective agent against ischemic stroke insults through this indirect Nrf2 mechanism leading to neuroprotection.

The permanent distal middle cerebral artery occlusion (pdMCAO) mouse model generates highly reproducible ischemic cortical lesion, inducing a definable behavioral deficit that closely mimics human ischemic stroke. It is therefore believed to be one of the most predictable and potentially useful stroke translational models, allowing us to look at longer time points with most high survival rate (Carmichael, 2005; Dorr et al., 2007; Doyle and Buckwalter, 2014). Different from histological outcome assessment, it is usually difficult to accurately estimate the behavior deficit in stroke animal models, especially the mild or moderate behavioral impairment in mice (Khaing et al., 2012). Till now, behavior tests were mostly applied to assess severe sensorimotor deficits, and few were reported to provide reliable and accurate evaluation for mild sensorimotor deficits caused by focal ischemic stroke.

In the present study, we hypothesized that the standardized Ginseng extract pretreatment protects against acute sensorimotor deficits and improve functional recovery after ischemic stroke potentially through the Nrf2 pathway; preferential alteration of the spatiotemporal reactive astrogliosis is associated with such effect. Accordingly, we had five goals in this study: (1) to establish a novel method that can accurately evaluate mild sensorimotor deficits in ischemic stroke mouse models; (2) to evaluate the effects of Ginseng pretreatment on acute sensorimotor deficits and anatomical outcomes and functional recovery following pdMCAO and determine whether these effects are lost in Nrf2^−/−^ mice; (3) to demonstrate the contribution of Nrf2 in the effects of Ginseng pretreatment by examining the induction of Nrf2 downstream target cytoprotective and antioxidative proteins; (4) to assess the contributions of the spatiotemporal reactive astrogliosis and microgliosis in the Ginseng effects in the acute phase of ischemic injury; and (5) to provide the *in vivo* evidence addressing the functional contribution of Nrf2 and its effects on reactive gliosis after focal permanent stroke.

## Materials and Methods

### Animals

All procedures on animals were performed according to the NIH Guide for the Care and Use of Laboratory Animals and approved by the University of Florida Institutional Animal Care and Use Committee. The Nrf2^−/−^ mice were generated as previously described (Itoh et al., 1997; Wang et al., 2011; Leonardo et al., 2013). Mice were kept on a standard 12 h light/12 h dark cycle with free access to food and water. Cohorts of matched wildtype (WT) and Nrf2^−/−^ C57BL/6 male mice between 10 and 18 weeks of age were used for this study. (1) Cohort 1: Twenty WT mice were used for the establishment of long-term sensorimotor assessment; (2) Cohort 2: Forty-five WT mice and 46 Nrf2^−/−^ mice were used for long-term sensorimotor evaluation of Ginseng pretreatment on ischemic stroke; (3) Cohort 3: Forty WT mice and 40 Nrf2^−/−^ mice were used for analysis of immunohistochemistry staining and Western blot. For each cohort in this study, mice were randomly distributed into groups of both genotypes and treatments.

### Experimental Ischemic Stroke Model

Ischemic stroke was induced by pdMCAO as described previously (Wang et al., 2011; Llovera et al., 2014). Each mouse was initially anesthetized with 5% isoflurane and maintained with 2% isoflurane in an oxygen/air mixture during surgery. Artificial tear ointment was applied to the eyes of each mouse for protection and lubrication. Under a stereo surgical microscope, a 0.8–1 cm incision between the right eye and ear was made, and then the temporal muscles were dissected bluntly and retracted outwards to visualize the temporal bone. To expose the distal MCA, a craniotomy was performed and a piece of 2 mm^2^ bone above the artery was carefully removed with a micro forceps. The distal MCA branches were occluded permanently using a bipolar electrocoagulation forceps (Bovie Medical Corp., Clearwater, FL, USA). The occlusion was checked 30 s later, and, if needed, the coagulation was repeated to achieve complete occlusion. The temporal muscles were repositioned and the skin incision was closed with wound clips. Body temperature was maintained at 37°C by a thermostatically controlled heating pad during surgery, and mice were allowed to recover in a temperature- and humidity-controlled chamber after surgery. The sham-operated (sham) control mice were subjected to the same surgery procedure, except that the distal MCA was not occluded. Mice that died during surgery or experiments were excluded from the final analyses. No apparent evidence of hemorrhages was observed during or after surgery. Mice that died during surgery or experiments were excluded from the final analysis. As for all of our animal colonies, fresh food and water were provided to the animal *ad libitum*.

### Ginseng Administration

The standardized Korean Red Ginseng extract is a red powder and is water soluble that is prepared and standardized as previous reports (Kim J. et al., 2008; Kim Y. T. et al., 2008). WT and Nrf2^−/−^ mice in cohort 2 received a once-daily gavage administration of either Ginseng at a concentration of 100 mg/kg in double-distilled water or vehicle during the morning hours (8–10 am) for 7 days prior to pdMCAO (Yan et al., 2013; Wang et al., 2014). During the whole experiment, mice were weighted before and after surgery as an indicator of their general well-being.

### Behavioral Testing

A battery of behavior tests, including open field, cylinder and corner tests, was designed for the long-term functional assessment of ischemic stroke mice, especially for sensorimotor deficits (Brooks and Dunnett, 2009; Schaar et al., 2010; Balkaya et al., 2013a). All mice were subjected to behavior experiments on indicated days before and after pdMCAO or sham surgery. To minimize the possibility that the behavioral response would be affected by previous task, the least stressful test was conducted at the early stage and the invasive test was performed at the late stage. Therefore, each mouse was first subjected to an open field test and then a cylinder; test followed by a corner test with at least 1 h intervals between tests. Experimenters for tests and analyses were blind to group assignment, treatment and genotype.

### Open Field Test

The open field paradigm was used for assessing the general spontaneous locomotor activity of mice in a novel environment with an automated video tracking system (MED Associates, St. Albans, VT, USA; Singh et al., 2013). Mice were individually placed in a plastic chamber and allowed to explore the arena for 30 min. Animal movement was monitored with a computer-linked camera. The total ambulatory distance for each mouse was detected by the tracking system. The chamber was cleaned with 70% ethanol and air dried between tests.

### Cylinder Test

The cylinder test was used to measure the unilateral deficits in voluntary forelimb use for assessing the sensorimotor impairment (Fleming et al., 2013; Llovera et al., 2014). A transparent acrylic glass cylinder, 8 cm in diameter and 25 cm in height, was placed on top of a piece of glass with a supporting frame. A mirror was placed at a 30- to 45-degree angle beneath the glass. A camera was placed in front of the mirror and able to view the entire space inside the cylinder by adjusting the angle of the mirror (to obtain an optimal video). A mouse was placed in the cylinder and recorded for 6 min. Quietness and very little movement from the experimenter are important to avoid any potentially freezing behavior. The cylinder and glass were cleaned with 70% ethanol and allowed to dry between mice. Of note, the general activity of ischemic mice might be decreased during the acute period after pdMCAO. At least 10 times of full rearings and 20 times of forelimb contacts are needed for an increased accuracy of analyzing. Rearing without any forelimb contact was not incorporated. Frame-by-frame analysis by the VideoLAN Client media player software was used for forelimb contacts. To assess the voluntary forelimb use, we counted and analyzed the following parameters: (1) total forelimb use (contralateral side to ischemic lesion, %), which is the total number of all forelimb contacts on the cylinder wall while full rearings were counted; (2) first contact events (with both forelimbs, left forelimb, or right forelimb), which is when the first contact of a mouse on the cylinder wall happened with both forelimbs or single forelimb (left/right) during one full rearing period; and (3) both forelimbs use events (%) or single forelimb use events (with left/right, %), which is when the mouse contacted the cylinder wall with both forelimbs or always used the same forelimb during one full rearing period. The definitions for other parameters: full rearing, which is when mouse rears up on two hind legs only; forelimb use is defined as the placement of the whole palm on the wall of cylinder to support the body in full rearing instances; and the contact with both forelimbs or single forelimb (left/right), which is when the mouse contacts the cylinder wall with both forelimbs simultaneously (the interval of both forelimbs contacts is no more than three frames at 29 frames per second) or with a single forelimb.

The calculations of above parameters are: (1) total forelimb use (left, %) = total number of contacts with left side of forelimb/total number of contacts with each side of forelimb × 100; (2) first contact events (with both forelimbs, left forelimb, or right forelimb) = total number of first contact events (with both forelimbs, left forelimb, or right forelimb)/total number of full rearing with any forelimb contact × 100; and (3) both forelimbs use events (%) and single forelimb use events (with left/right, %) = total number of both forelimbs use events or single forelimb (left/right) use events/total number of full rearing with any forelimb contact × 100.

### Corner Test

The corner test was performed to determine overall sensorimotor and postural asymmetries (Lubjuhn et al., 2009; Balkaya et al., 2013b; Langhauser et al., 2014; Shen et al., 2014; Liu et al., 2016). A mouse was gently placed between two non-transparent glass boards (30 cm × 20 cm × 0.5 cm each) set at a 30° angle. The mouse faced about 5 cm away from the corner. When the mouse explored closer into the corner, their vibrissae on both sides were simultaneously stimulated. The mouse would stand on two legs, placing its forepaws against the wall, and then would turn back to the open side of the corner. The normal mouse turned back using its left or right side at the same rate spontaneously, whereas the pdMCAO-induced ischemic stroke mouse was inclined to turn to the contralateral side of the lesion (left; Lubjuhn et al., 2009; Balkaya et al., 2013b). Each mouse performed 10 trials with at least 5 s intervals between each trial. The results were expressed as the percentage of left turn in 10 trials. The turning without any rearing or forepaw placement against the wall was excluded from the analysis.

### Measurement of Infarct Volume

The infarct volume was assessed by either 2,3,5-triphenyltetrazolium chloride (TTC) or cresyl violet staining on day 1 and day 3 after ischemic stroke, respectively. The brains were quickly removed after euthanasia and sliced into 1 mm coronal sections using a brain matrix. Slices were incubated for 20 min at 37°C in 2% TTC (Sigma-Aldrich) dissolved in phosphate-buffered saline (PBS, pH 7.4) for vital staining (Bederson et al., 1986). Each section was scanned, and the infarct area was analyzed using ImageJ software (National Institutes of Health, Rockville, MD, USA). Brains were cut into 30-μm-thick coronal slices on a Leica rotary microtome cryostat. Every tenth section throughout the infarct-containing region was stained with cresyl violet as previously described (Zechariah et al., 2010; Manwani et al., 2014). Sections were scanned using ScanScope CS (Aperio Technologies, Inc., Vista, CA, USA), and the border between infarcted and healthy tissue was outlined with ImageScope software (Aperio Technologies). Lesion areas from various rostrocaudal brain levels were integrated for lesion volume analysis. To account for brain swelling due to edema following ischemic injury, the corrected total infarct volume (%) was calculated as previously described: corrected infarct volume (%) = [volume of contralateral hemisphere − (volume of ipsilateral hemisphere − volume of infarct)]/volume of contralateral hemisphere × 100 (Swanson and Sharp, 1994; Langhauser et al., 2014; Gu et al., 2016).

### Histology and Immunohistochemistry

At indicated time points, mice were anesthetized and transcardially perfused with PBS (pH 7.4) followed by 4% paraformaldehyde (PFA, pH 7.4) in PBS. The brains were collected, post-fixed and cryoprotected in 30% (w/v) sucrose and cut into 30-μm-thick coronal slices on a Leica rotary microtome cryostat. Immunostaining was performed using standard protocols. Sections were washed and then treated with 3% H_2_O_2_ (V/V) for 15 min at room temperature to remove endogenous peroxidase activity. After washing and blocking with 2% normal horse serum (V/V), sections were incubated with primary antibodies overnight at 4°C. The following primary antibodies were used: rabbit polyclonal glial fibrillary acidic protein (GFAP; 1:3000; DAKO, Carpinteria, CA, USA), rabbit polyclonal ionized calcium-binding adapter protein 1 (Iba1; 1:5000, Wako Bioproducts, Richmond, VA, USA), mouse monoclonal glutamine synthetase (GS; 1:3000; EMD Millipore, Billerica, MA, USA), and rabbit polyclonal aquaporin 4 (AQP4; 1:3000; EMD Millipore). On the following day, sections were washed and incubated with appropriate secondary antibodies and visualized with 3,3-diaminobenzidine (DAB) substrate (Vector Laboratories). We counted the total numbers of GFAP-positive astrocytes, GS immunointensity, APQ4 immunointensity and the total number of Iba1-positive cells in indicated regions. The percentages of GFAP-positive reactive astrocytes were also measured, featured by hypertrophic cell bodies and intensely stained processes. Three consecutive sections from each brain were analyzed and provided a single value for this mouse. The images were captured using ScanScope CS and analyzed using ImageScope Software (Aperio Technologies). To avoid potential bias, two independent analyses were performed by two trained observers blinded to pretreatment and genotype.

### Western Blot

Western blot analyses were performed as previously described (Wang et al., 2011). Animals were anesthetized and sacrificed by cervical dislocation. The brain tissue was immediately harvested and stored at −80°C. The tissue dissected from mouse brain was homogenized in RIPA buffer. After centrifuging at 12,000 rpm for 5 min, supernatant was collected as the total cell lysate. Protein concentration was measured using the BCA Protein Assay Kit from Thermo Fisher Scientific. Supernatant was separated on 4%–15% polyacrylamide Tris-HCl gradient gels (BioRad, Hercules, CA, USA) and transferred to PVDF membranes. The integrated optic density of the immunoreactive band was quantified with NIH ImageJ software. The primary antibodies used were: rabbit polyclonal superoxide dismutase 2 (SOD2; 1:2000; EMD Millipore), rabbit polyclonal glutathione peroxidase 1 (GPx1; 1:1000; Abcam, Cambridge, MA, USA), goat polyclonal NAD(P)H:Quinone Oxidoreductase 1 (NQO1; NQP1; 1:1000; Abacam), rabbit polyclonal heme oxygenase 1 (HO1; 1:1000; Enzo Life Sciences, Farmingdale, NY, USA) and mouse anti-actin (1:5000, EMD Millipore).

### Statistical Analysis

Statistical analysis was performed using SAS-JMP software (SAS Institute, Cary, NC, USA) with assistance from a biostatistician. The number of mice in independent experiments is stated in each figure or figure legend. Behavioral tests were analyzed by Repeated Measures Linear Mixed Modeling to account for identified baseline and post-ischemic outcome differences between groups. Multi-group comparisons were carried out using one-way or two-way ANOVA followed by Bonferroni *post hoc* tests. All data were expressed as mean ± SEM. *P* < 0.05 was considered statistically significant.

## Results

### Optimizing a Long-Term Sensorimotor Assessment in a Mild Ischemic Stroke Mouse Model

**Figure 1:**
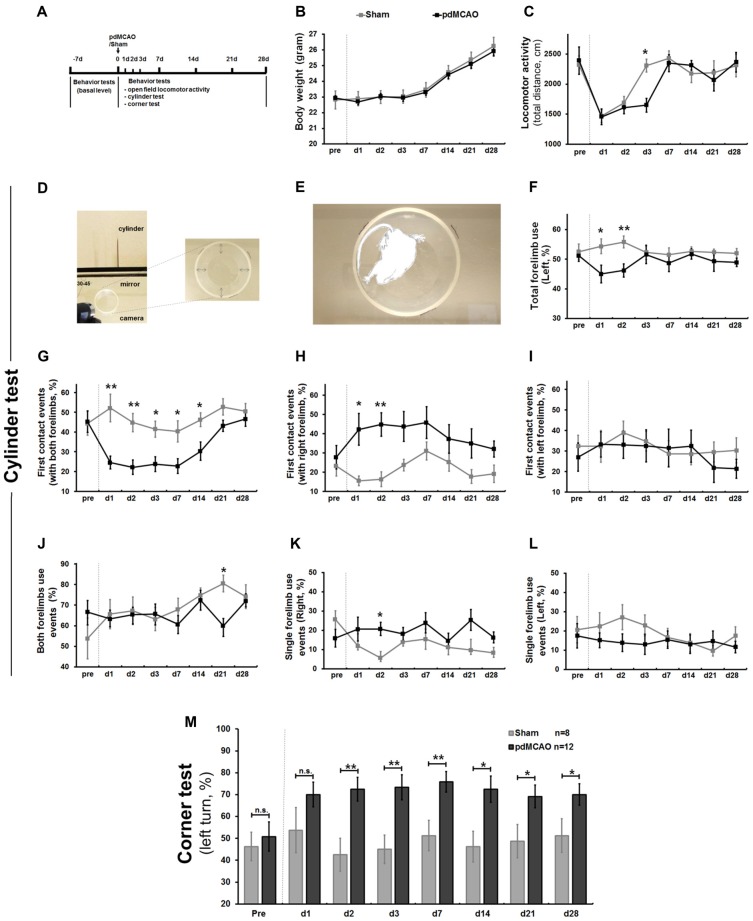
Establishment of long-term sensorimotor assessment in pdMCAO model mice over 4 weeks. **(A)** Scheme of the experimental design. **(B)** The pdMCAO mice did not exhibit significant weight change. **(C)** In the open field test, mice in both groups traveled similar distance at most time points except day 3 (*P* < 0.05) after surgery when compared with baseline measurements. **(D)** Schematic representation of the cylinder test paradigm. **(E)** Forelimbs explorations were assessed by traditional **(F)** and multiple new designed parameters **(G–L)**. Compared to Sham, the pdMCAO mice exhibited remarkable decrease in percentage of total forelimb (affected, contralateral to the ischemic lesion side, **F**) use on day 1 (*P* < 0.05) and day 2 (*P* < 0.01), but not at later time points. Most prominently, the post-ischemic mice displayed significant decrease of the first contact events rate (with both forelimbs, **G**) on day 1, 2, 3, 7, 14 and tendency of gradual recovery by day 28 (*P* > 0.05). No significant difference was detected in the first contact events (with right/left forelimb) at the late stage (day 3–28) of ischemic stroke **(H,I)**. No significant difference was revealed in both forelimbs use events **(J)** or single forelimb events **(K,L)** between ischemic stroke and control groups at most time points. **(M)** In corner test, post-ischemic mice displayed significant increase of left turn percentage (contralateral side) at most time points over 4 weeks compared to sham controls, except for day 1. *P < 0.05, **P < 0.01 in forelimb use compared to controls. Sham group, *n* = 8; pdMCAO group, *n* = 12. pdMCAO, permanent distal middle cerebral artery occlusion; Sham, sham-surgery; d, day.

To optimize a method that can accurately evaluate sensorimotor deficits in pdMCAO mice model, a typical mild ischemic stroke model, we performed a behavioral test battery over 4 weeks (Figure 1A). Weight change dynamics, in comparison to baseline body weight, appears to be a strong indicator of poor post-ischemic outcome (Scherbakov et al., 2011). Compared to sham surgery mice, pdMCAO mice did not exhibit significant weight change (Figure 1B). As shown in Figure 1C, pdMCAO-induced ischemic damage dramatically decelerated the recovery tendency of locomotor activity at the earlier stages (1–7 days; day 3: *P* < 0.05), but not at the later stages (7–28 days). No animal died during these experiments. These results indicated that pdMCAO led to the reduction of early-stage locomotor activity without affecting weight change dynamics.

Sensorimotor dysfunction, the earliest and most prominent symptom of pdMCAO-induced ischemic mice, was evaluated by analyzing voluntary forelimb use in a cylinder test (Figures 1D–L). We designed multiple parameters to characterize sensorimotor asymmetry. First, we analyzed the total number of contralateral forelimb use, the only parameter used in traditional methods. Consistent with previous reports, the ischemic mice exhibited a marked decrease on days 1 and 2 compared to shams (*P* < 0.05 and *P* < 0.01, respectively), but not at the later time points (Figure 1F; Llovera et al., 2014). Second, we analyzed the first contact events (with both forelimbs, left forelimb or right forelimb). The ischemic mice were inclined to use the ipsilateral forelimb, a tendency which consequently led to lower rate of use of both forelimbs simultaneously, particularly reflected by first contact events rate in full rearings. Most prominently, the ischemic mice displayed a significant decrease in the first contact events with both forelimbs from day 1 to day 2 and from day 3 to day 14 (*P* < 0.01 and *P* < 0.05, respectively); this difference was not detected between days 21 and 28 when compared with shams (*P* > 0.05, Figure 1G). In contrast, unexpectedly, no significant difference was detected in the first contact events with right/left forelimb at the late stage, although we presumed that these parameters might be sensitive (day 3–28, Figures 1H,I). Finally, we analyzed single forelimb use events and both forelimbs use events to further identify possible parameters. However, we did not observe any significant difference at most time points (Figures 1J–L). These data indicated that pdMCAO induced ischemic injury led to remarkable unilateral deficits in voluntary forelimb uses over 4 weeks with a tendency of long-term sensorimotor recovery. This new designed parameter, first contact events with both forelimbs, appeared to be more sensitive and reliable when reflecting either severe or mild sensorimotor deficits compared to others (Figure 1G). This would be valuable for long-term functional evaluation in ischemic mice after pdMCAO. Consistent with previous report (Lubjuhn et al., 2009), mice displayed similar preferential turnings to each side at baseline (Figure 1M), and sham-surgery did not lead to any inclination over 4 weeks. In the corner test, ischemic injury resulted in prominent increases in left turn percentage (contralateral) on days 2–28 compared with sham counterparts (Figure 1M). In contrast to a previous report, no recovery tendency in this parameter was detected after pdMCAO, indicating that the corner test is comparably less sensitive in detecting the alteration of sensorimotor deficits after pdMCAO (Lubjuhn et al., 2009).

### Ginseng Pretreatment Attenuates Acute Ischemic Sensorimotor Deficits and Promotes Long-Term Recovery, Whereas Loss of Nrf2 Abolishes Such Protection

**Figure 2:**
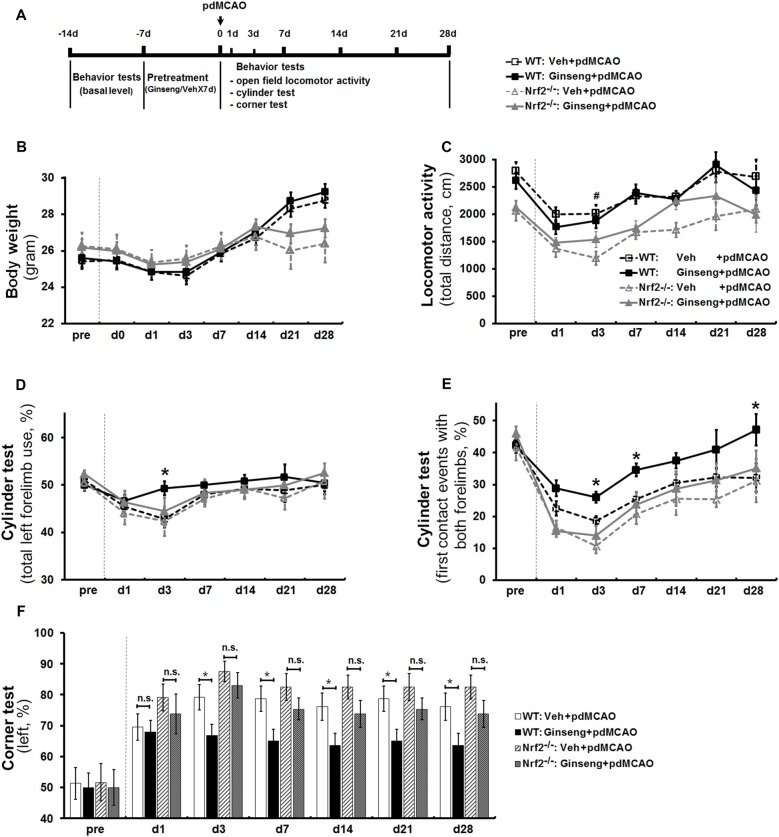
Ginseng pretreatment attenuates sensorimotor deficits and promotes long-term functional recovery over 4 weeks after ischemic stroke, but not in Nrf2^−/−^ mice. **(A)** Experimental design. Ginseng pretreatment did not affect the body weight change **(B)** or locomotor activity **(C)** at any indicated time points compared to vehicle-pretreated controls of both genotypes. However, Ginseng pretreatment protected against sensorimotor deficits and improved the recovery over 4 weeks after pMCAO in wildtype (WT) mice, but failed to do so in Nrf2^−/−^ mice, a finding reflected by significantly increased percentages of both total forelimb use and first contact events with both forelimbs on day 3 after pdMCAO (*P* < 0.05, **D,E**, respectively). At the late stage (3–28 days), Ginseng pretreatment dramatically promoted the long-term sensorimotor recovery (days 7 and 28: *P* < 0.05, **E**) to baseline level. **(F)** The left turn percentage in corner test. *n* = 17–23 per group. *P < 0.05, ^#^*P* < 0.05. d, day.

Based on this optimized method for the cylinder test, with another cohort of mice, we investigated whether Ginseng pretreatment is protective against ischemic damage and if this effect is lost in the Nrf2-deficient mice (Figure 2A). After pdMCAO, there was no significant difference in body weight observed among the groups except on day 21 and day 28 (Figure 2B). In the open field test, post-ischemic mice in all groups traveled apparently less distance in 30 min on day 1 after pdMCAO when compared with baseline measurements (Figure 2C). Ginseng pretreatment did not affect the locomotor activity at any indicated time points compared to vehicle-pretreated controls of both genotypes. Between vehicle-pretreated groups, the Nrf2^−/−^ mice displayed relatively lower locomotor activity. Three mice of both genotypes died only during the acute phase of the surgeries for technical issues and were not included in the stroke cohorts.

In cylinder test, baseline behavior levels were similar in all groups. At the acute stage of ischemic stroke (0–3 days), Ginseng pretreated WT mice exhibited remarkable attenuated sensorimotor deficits compared to vehicle-pretreated WT controls, reflected by significantly increased percentages of both total forelimb use and first contact events with both forelimbs on day 3 after pdMCAO (*P* < 0.05, Figures 2D,E, respectively). At the later stages (3–28 days), Ginseng pretreatment dramatically promoted the long-term functional recovery to baseline level (day 7: *P* < 0.05; day 28: *P* < 0.05; Figure 2E). These results indicated that Ginseng protected against acute ischemic sensorimotor deficits and promoted long-term functional recovery. In contrast, this beneficial effect was not detected in Nrf2^−/−^ mice at all indicated time points. In addition, loss of Nrf2 exacerbated the early stage of functional deterioration but did not affect the recovery tendency of ischemic stroke (day 1: *P* < 0.05). The corner test revealed that Ginseng pretreatment significantly preserved the sensorimotor function from day 3 to day 28 in WT but not in Nrf2^−/−^ mice (Figure 2F). In addition, Nrf2 deficiency did not lead to significant differences between vehicle-pretreated groups.

### Ginseng Pretreatment Protects Against the Infarct Volume Expansion of Ischemic Brain Lesion in WT, But Not in Nrf2^−/−^ Mice

**Figure 3:**
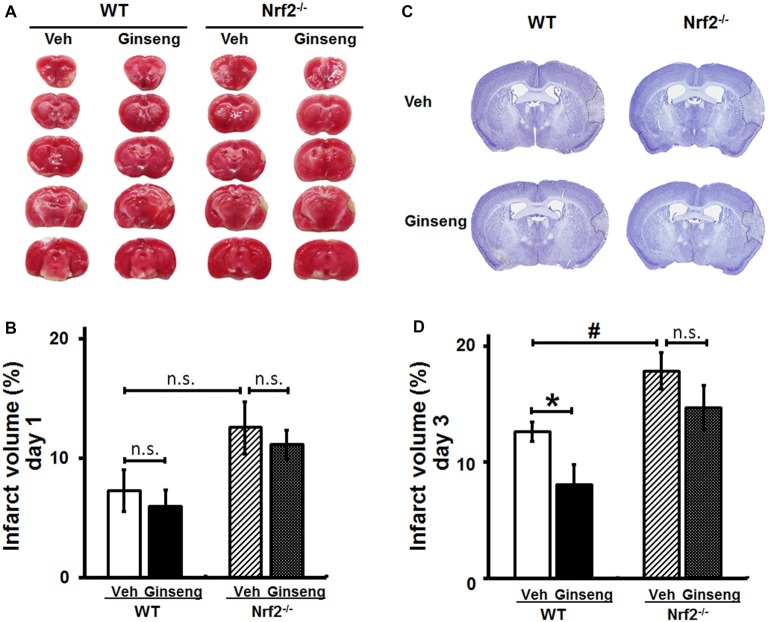
Ginseng pretreatment attenuates the expansion of infarct volume following experimental ischemic stroke in WT mice, but not in Nrf2^−/−^ mice. The effect of Ginseng pretreatment on infarct volume on day 1 and 3 after ischemic stroke was determined by either TTC **(A,B)** or cresyl violet **(C,D)** staining in both WT and Nrf2^−/−^ mice. The representative photographs and quantification of infarct volume in serial coronal sections showed that, on day 1 **(A,B)**, there was no significant difference between either treatments or genotypes. However, on day 3 **(B,D)**, Ginseng pretreatment significantly reduced the infarct volume in WT mice (*P* < 0.05), but not in Nrf2^−/−^ mice, while Nrf2 deficiency also increased the infarct size (*P* < 0.05) in vehicle-pretreated groups. Statistical analysis was performed using two-way ANOVA with Bonferroni post-tests. *n* = 5–6 per group. *P < 0.05, ^#^*P* < 0.05. TTC: 2,3,5-triphenyl tetrazolium chloride.

Functional deficits caused by acute ischemic stroke are often associated with the extent of histological damage in affected brain regions. Thereafter, with the third cohort of mice, we examined whether Ginseng pretreatment could protect against acute ischemic lesion size and whether Nrf2 contributes to this process. Consistent with the behavioral findings above (Figure 2), Ginseng pretreatment remarkably attenuated infarct volume on day 3 but not on day 1 of the acute stage in WT mice after ischemia onset (day 3: 8.03 ± 1.74% vs. 12.62 ± 0.81%, *P* < 0.05; day 1: 5.94 ± 1.38% vs. 7.26 ± 1.55%, *P* > 0.05; Figures 3A–D). However, such neuroprotective effect was not present in Ginseng pretreated Nrf2^−/−^ mice when compared to vehicle-pretreated Nrf2^−/−^ mice (day 1: 11.12 ± 1.26% vs. 12.54 ± 2.17%, *P* > 0.05; day 3: 14.66 ± 1.93% vs. 17.84 ± 1.57%, *P* > 0.05). In contrast, compared to vehicle-pretreated WT controls, vehicle-pretreated Nrf2^−/−^ mice group displayed significantly severe infarct volume on day 3 but not on day 1 (*P* < 0.05 and *P* > 0.05, respectively) after pdMCAO. These results indicated that Ginseng pretreatment protected against ischemic lesion, an effect which was diminished by the loss of Nrf2, suggesting the potential involvement of Nrf2 activation in the neuroprotection of Ginseng pretreatment.

### Ginseng Pretreatment Enhances Expression Levels of Nrf2 Downstream Cytoprotective Proteins in WT Mice, But Not in Nrf2^−/−^ Mice

**Figure 4:**
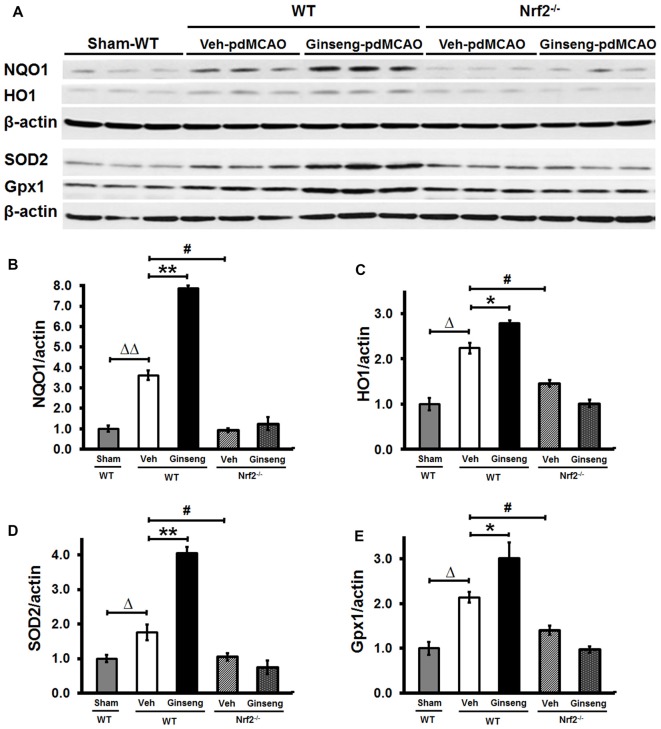
Ginseng pretreatment improves expression levels of Nrf2 downstream cytoprotective proteins in WT mice but not in Nrf2^−/−^ mice at 24 h after pdMCAO. **(A)** Western Blot of HO1, NQO1, SOD2 and GPX1 protein expressions on day 1 after pdMCAO in peri-infarct tissue of different groups. **(B–E)** Quantifications of above markers in **(A)**. Statistical analyses were carried out using one-way or two-way ANOVA. Data are presented as mean ± SEM. *n* = 4–5 per group. *p < 0.05, **p < 0.01, ^#^*p* < 0.05, ^Δ^*p* < 0.05, ^ΔΔ^*p* < 0.01. HO1, Heme oxygenase 1; NQO1, NAD(P)H:Quinone Oxidoreductase 1; SOD2, superoxide dismutase 2; GPx, glutathione peroxidase.

To demonstrate whether Nrf2 pathway activation plays a crucial part in the neuroprotective effects of Ginseng pretreatment against ischemic damage, we measured expression levels of Nrf2 downstream cytoprotective and antioxidative target proteins including HO1, NQO1, SOD2 and Gpx1 at the early stage of ischemic stroke (Figures 4A–E). Western blot analyses of peri-infarct tissue samples showed that Ginseng pretreatment significantly enhanced expression levels of HO1, NQO1, SOD2 and Gpx1 in WT mice, but not in Nrf2^−/−^ mice on day 1 after pdMCAO compared to vehicle controls. Additionally, Nrf2 deficiency significantly reduced expression levels of the markers above in vehicle-pretreated groups. These results by contrasting the protein expression pattern of Nrf2 downstream target genes between WT and Nrf2^−/−^ mice confirmed the unique role of Nrf2 activation in pdMCAO and further support that the Nrf2 pathway, at least partially, contributes to the beneficial efficacy of Ginseng pretreatment against ischemic damage.

### Ginseng Pretreatment Attenuates Spatiotemporal Reactive Astrogliosis to Ischemic Injury in WT Mice, But Not in Nrf2^−/−^ Mice

**Figure 5:**
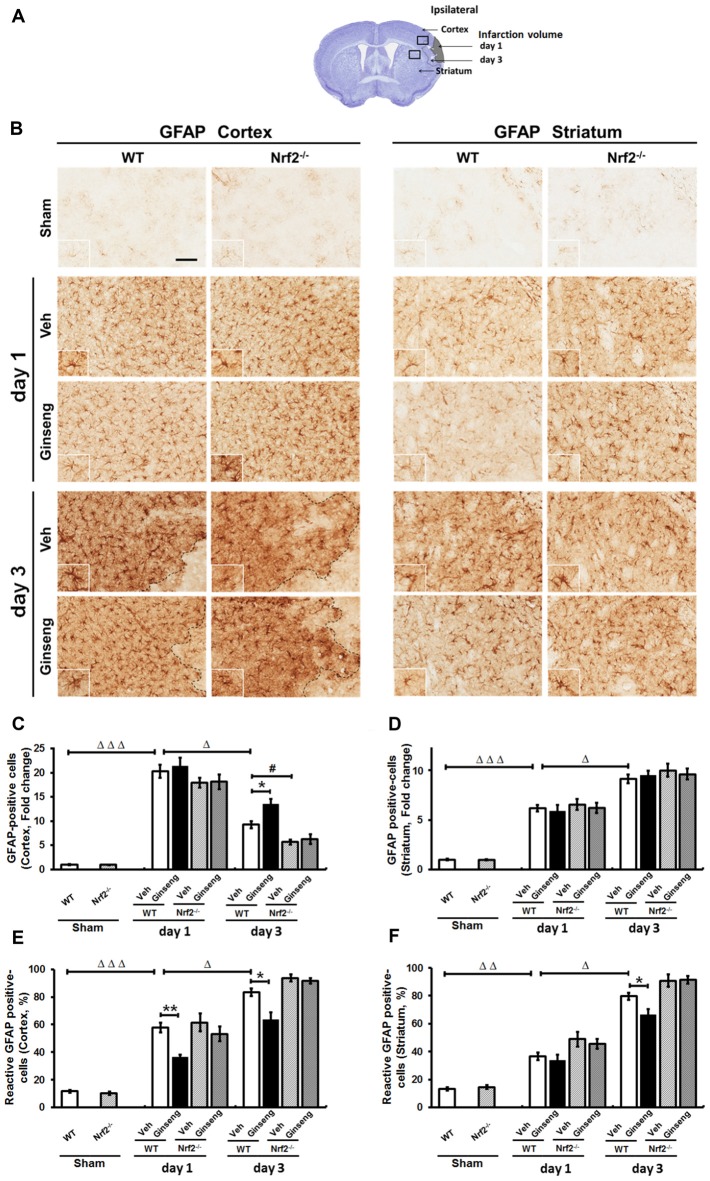
Ginseng pretreatment attenuates spatiotemporal reactive astrogliosis induced by pdMCAO.** (A)** Schematic representation of infarct area on day 1 and day 3 after pdMCAO; and open squares indicate the area in the cortex and striatum used for micrographic examination. **(B)** The representative photographs of GFAP-positive astrocytes in the ipsilateral cortex and striatum. Scare bar: 100 μm. Quantifications of the total number of GFAP positive astrocytes and the percentage of reactive astrocytes in the ipsilateral cortex **(C,D)** and striatum **(E,F)**. In cortex, compared with Veh-pretreatment WT group, Ginseng pretreatment did not affect the dramatic increase on day 1, but significantly preserved pdMCAO induced loss of the total number of astrocytes on day 3 in WT group **(C)**, whereas the percentages of reactive astrocytes were significantly lower in Ginseng pretreated WT group compared to Veh-pretreated WT group on both day 1 and day 3 **(D)**. Nrf2 absence exacerbated the astrocytes loss on day 3 after pdMCAO. However, in striatum, Ginseng pretreatment did not affect the increase of the total number of astrocytes **(E)**, but only reduced the percentage of reactive astrocytes on day 3 **(F)**. Statistical analysis was performed using two-way ANOVA with Bonferroni post-tests. *n* = 4–5 per group. *P < 0.05, **P < 0.01, ^#^*P* < 0.05, ^Δ^*P* < 0.05, ^ΔΔ^*P* < 0.01, ^ΔΔΔ^*P* < 0.001. GFAP: glial fibrillary acidic protein.

Reactive astrogliosis refers to the morphology, proliferation, gene expression and biochemical changes in reactive astrocytes in response to various CNS insults, having diverse and crucial functions in the pathogenesis of ischemic brain damage (Pekny and Pekna, 2014). We next examined whether reactive astrogliosis contributes to the neuroprotection of Ginseng by analyzing the changes of the spatiotemporal profile of astrocytes in the peri-infarct areas by using immunostaining of GFAP (Figure 5). In the shams (Figures 5A,B, top panels), astrocytes tiled the entire cortex and striatum in a regular distribution pattern in both WT and Nrf2^−/−^ mice, most of exhibiting a nonreactive phenotype with small soma and fine processes. Ischemic injury triggered reactive astrocyte proliferation, which was indicated by the increased number of astrocytes at the peri-infarct area. A larger proportion of astrocytes were activated, featuring hypertrophic cell bodies and highly stained processes. In the cortex (Figures 5A–D), Ginseng pretreatment did not affect the total number of astrocytes in both genotypes, but rather exhibited a prominently smaller proportion of reactive astrocytes in WT mice on day 1 after pdMCAO compared to the vehicle-pretreated control (*P* > 0.05 and *P* < 0.01, respectively). On day 3, Ginseng pretreatment preserved more astrocytes and smaller proportions of reactive astrocytes in WT mice, but not in Nrf2^−/−^ mice (*P* < 0.05, respectively), while the Nrf2 deficiency significantly exacerbated the astrocyte loss (*P* < 0.05). Additionally, much more degenerated astrocytes with breakdown cell bodies were found in Nrf2^−/−^ mice groups than WT mice groups. These results indicated that the Nrf2 activation from Ginseng pretreatment may attenuate the early stage of progression of reactive astroglosis that helped prevent the expansion of ischemic infarct volume (day 1–3, Figure 3). This spatiotemporal pattern of reactive astrogliosis in the cortex correlated well with that of ischemic damage. However, in the striatum, Ginseng pretreatment only reduced the proportion of reactive astrocytes on day 3 after pdMCAO (Figures 5E,F).

**Figure 6:**
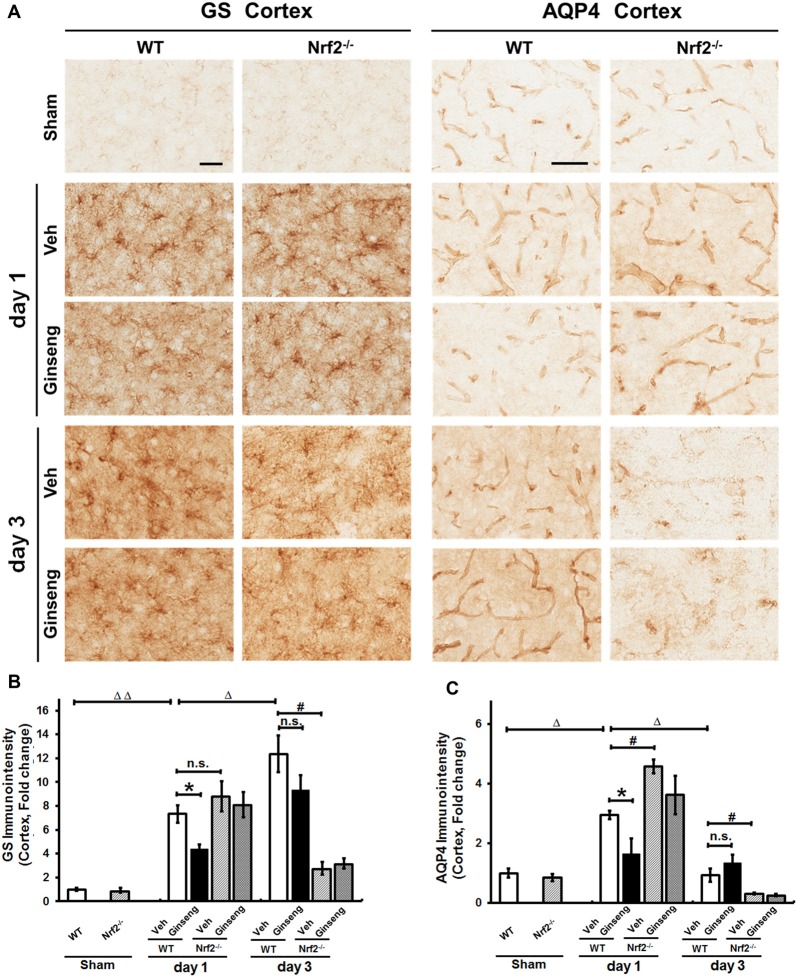
Ginseng pretreatment abates the disturbance of glutamate metabolism and water homeostasis in acute stage of ischemic stroke. **(A)** The representative photographs of GS and AQP4 in the ipsilateral cortex. Scare bar: 50 μm. The GS-positive **(B)** and AQP4 **(C)** signals in the ipsilateral cortex were quantified. Compared with Veh-pretreatment group, Ginseng pretreatment attenuates the sharp increase of GS expression on day 1 (*P* < 0.05) but not day 3 after pdMCAO, whereas this effect is absent in Ginseng pretreated Nrf2^−/−^ group. Additionally, Nrf2 absence exacerbated ischemic injury induced GS loss of on not day 1 but day 3 (*P* < 0.05) **(B)**. Ginseng pretreatment also significantly reduced the remarkable increase of AQP4 on day 1 (*P* < 0.05), but not on day 3 after pdMCAO **(C)**. No difference was detected between both Nrf2^−/−^ groups at any time points. Veh-pretreated Nrf2^−/−^ group exhibited strikingly higher AQP4 expression level on day 1 (*P* < 0.05) and underwent similar sharp decrease compared with WT control group. Statistical analysis was performed using two-way ANOVA with Bonferroni post-tests. *n* = 4–5 per group. n.s.: no significance; *P < 0.05, ^#^*P* < 0.05, ^Δ^*P* < 0.05, ^ΔΔ^*P* < 0.01. GS, glutamine synthetase; AQP4, Aquaporin 4.

Astrocytes control extracellular levels of glutamate through a glutamate-glutamine shuttle via GS and maintain brain water homeostasis through AQP4. We examined whether astrocytic functions on glutamate clearance and water transport were affected in ipsilateral cortex. GS immunostaining signals were detected at both cell bodies and processes of astrocytes throughout cortex (Figure 6A). Compared with vehicle pretreatment (Figure 6A), Ginseng pretreatment reduced the sharp increase in GS immunostaining density on day 1 (*P* < 0.05) but not day 3 after pdMCAO in WT mice; this effect was absent in Ginseng pretreated Nrf2^−/−^ group. Ginseng pretreatment reduced the rapid increase of AQP4 on day 1 (*P* < 0.05) but did not significantly affect the loss of AQP4 on day 3 after pdMCAO in WT mice (Figure 6C). No difference was detected between both Nrf2^−/−^ groups on both day 1 and 3. In addition, Nrf2 deficiency increased AQP4 expression level on day 1 and exacerbated the deteriorations of GS and AQP4 expression levels on day 3 in vehicle-pretreated mice (*P* < 0.05, respectively, Figure 6B). These results indicated that Ginseng pretreatment attenuated astrocytic dysfunction in glutamate metabolism and water homeostasis in a spatiotemporal pattern that was absent in Nrf2^−/−^ mice, further supporting the crucial effect of Nrf2 activation on reactive astrogliosis in the context of pdMCAO-induced ischemic stroke.

### Microglial Activation May Not Be Involved in the Neuroprotection of Ginseng Pretreatment Against Ischemic Injury

**Figure 7:**
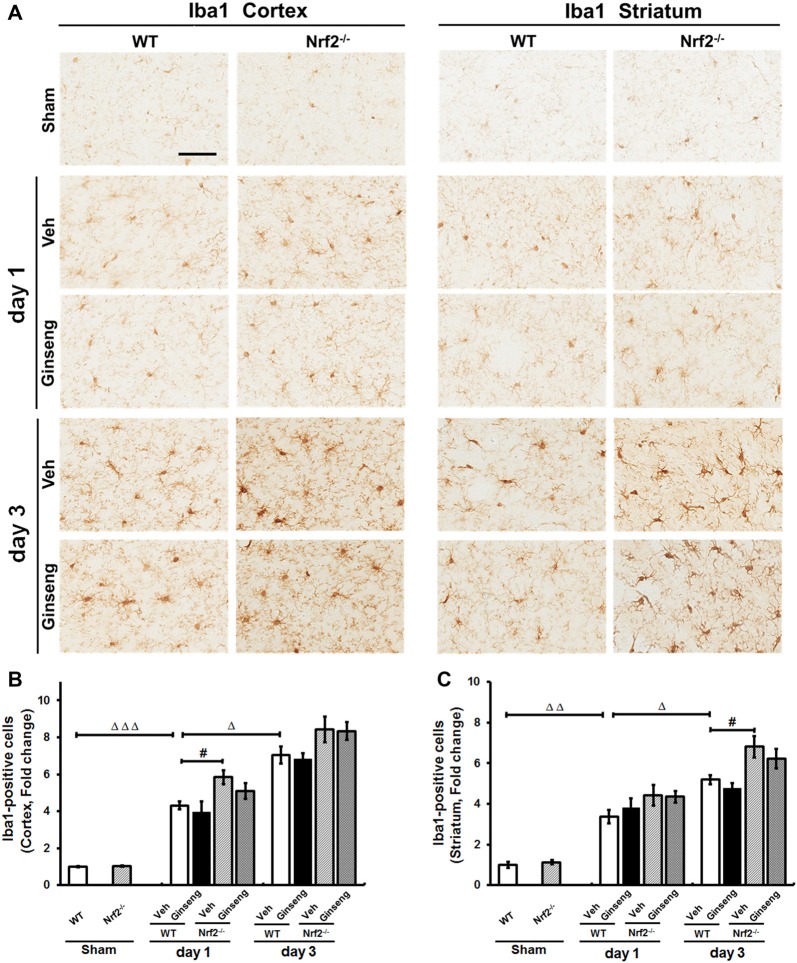
Ginseng pretreatment does not significantly affect the progression of microgliosis induced by pdMCAO. **(A)** The representative photographs of Iba1-positive microglia in the ipsilateral cortex and striatum. Scare bar: 50 μm. The Iba1-positive signal in the ipsilateral cortex **(B)** and striatum **(C)** were quantified. Ischemic injury induced activation of microglia (upregulated Iba1 immunoreactivity) on day 1 after pdMCAO, which lasted at least 3 days in both cortex and striatum **(B,C)**. Ginseng pretreatment had no gross effect on such microglia activation. Interestingly, Nrf2 absence exacerbated the microglial activation on day 1 in cortex and on day 3 in striatum (*P* < 0.05). Statistical analysis was performed using two-way ANOVA with Bonferroni post-tests. *n* = 4–5 per group. ^#^*P* < 0.05, ^Δ^*P* < 0.05, ^ΔΔ^*P* < 0.01, ^ΔΔΔ^*P* < 0.001. Iba1, Ionizing calcium-binding adaptor molecule 1.

Finally, we also investigated whether microglial activation contributes to the neuroprotection of Ginseng by Iba1 immunostaining. As shown in Figure 7, ischemic injury induced activation of microglia on day 1 after pdMCAO, which lasted at least 3 days in both cortex and striatum regions. However, no gross effect on microglia activation by Ginseng pretreatment was detected. Functional loss of Nrf2 exacerbated the microglia activation by Iba1 immunostaining on day 1 in cortex and on day 3 in striatum (*P* < 0.05). Our data indicated that, under our experimental condition, microglia activation would not have a substantial role in Ginseng’s neuroprotection against ischemic damage at indicated time points, while Nrf2 absence exacerbated the microglial activation.

## Discussion

In the present study, we optimized a method that can more accurately assess both severe and mild sensorimotor deficits in pdMCAO-induced ischemic stroke mice model. By using this method and Nrf2^−/−^ mice, we found that 7 days of Ginseng pretreatment dramatically attenuated acute sensorimotor deficits and promoted long-term recovery over 28 days after ischemic injury, which was accompanied by the reduction of acute brain lesion volume (36.37 ± 7.45% on day 3). Such neuroprotection was lost in Nrf2^−/−^ mice. Meanwhile, we found that Ginseng pretreatment significantly enhanced expression levels of Nrf2 downstream cytoprotective and antioxidative proteins including NQO1, HO1, SOD2 and GPx1 at the early stage of ischemic stroke onset in WT, and this was completely abolished in the Nrf2^−/−^ mice. Such Nrf2-mediated effects might attenuate spatiotemporal reactive astrogliosis, but they would not attenuate the microglia activation, that eventually contributed to the neuroprotection of Ginseng against ischemic damage. In addition, such insights concerning the role of Nrf2 deficiency in ischemic stroke progression induced by pdMCAO offered further understanding of the natural properity of Nrf2 in neuroprotection.

### Long-Term Sensorimotor Assessment in Ischemic Stroke Model Mice

Functional assessment is a key component for testing the efficacy of potential therapeutics in translational stroke research and sensitive behavior tests, especially in mice, are known to be challenging. The cylinder test was initially designed to assess the side-biased forelimb functional deficit in the Parkinson’s disease rat model, and then was extended to various rodent stroke models indicated by the rate of total forelimb use (contralateral side to brain lesion; Schallert et al., 2000; Iancu et al., 2005; Balkaya et al., 2013a). However, this traditional single-parameter method appeared to be not satisfyingly sensitive to mild sensorimotor deficits, the assessment of which is particularly important for long-term functional evaluation after ischemic stroke (Freret et al., 2009; Balkaya et al., 2013b). Due to the unilateral sensorimotor damage, the ischemic mice tended to use the ipsilateral forelimb instead of the contralateral forelimb, and consequently the habit of using both forelimbs simultaneously would be disrupted. Thus, we analyzed the first contact events—which forelimb(s) the mouse preferred to use first during all full rearings—to reflect subtle functional deficits. The parameter for the first contact events with both forelimbs rather than with the left or right forelimb appeared to be very sensitive to detect both severe and mild sensorimotor deficits. In addition, we considered that single or both forelimb(s) use events might be affected as well. However, no significant difference was detected. Consistent with previous reports of the corner test (Dorr et al., 2007; Balkaya et al., 2013a), our study showed that the pdMCAO mice exhibited dramatic long-term sensorimotor deficits, yet we observed no obvious recovery tendency. This indicated the lower sensitivity of sensorimotor deficits evaluation compared to the above new parameter in the cylinder test. Together, the first contact events (with both forelimbs) combined with the rate of total forelimb use in cylinder test provided the most sensitive and reliable assessment for both severe and mild sensorimotor deficits in pdMCAO model mice.

### Ginseng Pretreatment Protects Against Ischemic Stroke Potentially Through the Nrf2 Pathway

Ginseng has been shown to direct stimulation of cell defense mechanisms and alter specific antioxidant enzyme activities that are necessary for eliminating free radicals and reducing by-products of tissue peroxidation reactions (Gillis, 1997; Attele et al., 1999). The major active ingredients of standardized Ginseng extract contain most of its pharmacologic activity; most studies have shown that beneficial effects of Ginseng were achieved only by pretreatment (Wen et al., 1996; Kitts and Hu, 2000; Rastogi et al., 2014). Brain damage and functional impairment after cerebral ischemia is the result of the interaction between endogenous protective mechanisms and ischemic events that ultimately lead to neuronal death. Ginseng pretreatment is likely required to increase the intracellular concentration of bioactive compounds and antioxidant constituents or to increase a cascade involving an endogenous antioxidant system that potentially lessens the subsequent severe ischemic damage (Rastogi et al., 2014). Considering the molar ratio to neutralize free radicals, we believe the beneficial effects of Ginseng would be indirect through the Nrf2-indirect antioxidant pathway. To our knowledge, our study is the first to show the long-term neuroprotection of Ginseng pretreatment on ischemic stroke induced by pdMCAO. We did not detect the benefit of Ginseng pretreatment on the body weight dynamics and locomotor activity. However, Ginseng pretreatment strikingly protected against the deterioration of acute ischemic damage and promoted long-term sensorimotor functional recovery over the whole experimental duration. Activation of antioxidant pathways by Ginseng is particularly important for cytoprotection in brains with relatively weak endogenous antioxidant defenses. Under the condition of Nrf2 deficiency, the neuroprotective effects of Ginseng pretreatment were nearly eliminated in above measures at most time points, indicating the potential involvement of Nrf2 activation in the protection. It should be pointed out that there was another possibility. The beneficial outcomes of Ginseng on ischemic damage might through mechanism independent of Nrf2 activation, which was counteracted by the severe damage induced by Nrf2 absence. Thus, to confirm the functional importance of Nrf2 activation in Ginseng’ neuroprotection, Nrf2 downstream target markers including NQO1, HO1, SOD2 and GPx in peri-infarct tissue of cortex were measured at earlier-stage of ischemic stroke onset. Our data suggested that Ginseng pretreatment was correlated with remarkable induction of Nrf2 downstream target markers, including NQO1, HO1, SOD2 and Gpx in the cortex, indicating the importance of Nrf2 activation in Ginseng’s neuroprotection. Since currently there is no effective commercial Nrf2 antibody (Lau et al., 2013; Kemmerer et al., 2015), it limits the direct measurement of Nrf2 protection. Together, our findings showed that Ginseng pretreatment protected against the progression and the extent of acute ischemic damage, attenuated acute ischemic sensorimotor deficit, and promoted long-term functional recovery, which might through Nrf2 activation.

### Possible Roles of Nrf2 on Reactive Astroglosis in the Neuroprotection of Ginseng

Astrocytes are the most abundant cells in the CNS, sending out thin branches enwrapping neuronal synapses and covering adjacent brain capillaries within their anatomy domains. They play vital roles in maintaining brain homeostasis including brain energy metabolism, synaptic transmission regulation, potassium buffering, extracellular glutamate level control, and interstitial volume regulation. Neurons critically rely on astrocytes for their supportive properties and intrinsic protection in many CNS disorders, including ischemic stroke (Sims and Yew, 2017). In response to multiple CNS diseases or injuries, astrocytes display reactive properties associated with protective healing including progressive cellular hypertrophy, proliferation and migration. This consecutive, multistage and evolutionary conserved defensive reaction is termed as reactive astrogliosis, exhibiting altered expression of many genes, distinct morphological and functional features in CNS disease pathogenesis and the recovery process. The astrocytes also play a significant role in the glial scar formation following a focal tissue insult, and upregulation of various cellular markers, and other proliferating cell markers, have been described to be compromised in aged Sprague-Dawley rats as compared to younger ones (Popa-Wagner et al., 2007, 2018).

It is known that Nrf2-target genes are preferentially activated in astrocytes, which consequently have more efficient antioxidant defense and detoxification than neurons (Vargas and Johnson, 2009). Therefore, we investigated whether the Nrf2-mediated neuroprotective effects of Ginseng could be correlated with astrocytic activation. Nrf2-mediated neuroprotection by Ginseng attenuated the early-stage of development and progression of reactive astrogliosis in the peri-infarct cortex that conferred protective efficacy against ischemic brain lesion. Astrocytes transport more than 80% of extracellular glutamates via the glutamate-glutamine shuttle, sustaining glutamatergic synaptic transmission and protecting postsynaptic neurons from glutamate excitotoxicity, which is most important in an ischemic paradigm (Magistretti and Allaman, 2015; Pekny et al., 2016). GS is an ATP-dependent enzyme that is exclusively located in astrocytes, where it serves to maintain the glutamate-glutamine cycle. GS is particularly vulnerable to the oxygen- and nitrogen-centered radicals generated during ischemic stroke (Jeitner et al., 2015). Moreover, AQP4 is enriched in the perisynaptic astrocytic and perivascular processes, indicating key roles for water homeostasis in CNS. A strikingly dynamic change of AQP4 expression implies the coordination between water and glutamate passing through astrocytic membranes after ischemic injury. Our study showed that Ginseng pretreatment delayed the earlier stage of rapid increase and protected against the later stage of sharp decline in GS and AQP4 expressions levels after pdMCAO, which contributed to the maintenance of extracellular homeostasis. The coordinated regulation of AQP4 expression by Ginseng might play a compensatory role in reducing the intracellular hyperosmotic pressure initiated by impaired glutamate clearance in the astrocyte processes. Further investigation may be needed to demonstrate the exact pathophysiological roles of GS and AQP4 in the context of ischemic stroke. These findings revealed that Ginseng pretreatment attenuated the deteriorative disruption of astrocytic structural integrity and associated functions in glutamate clearance and water transport. The microglia also play crucial role in ischemic stroke and are an effective target for stroke therapy in animal models (Hu et al., 2014; Kim et al., 2015). In this study, according to the time frame of measurements, microglial activation appeared not to be involved in the neuroprotection of Ginseng in the peri-infarct area of both the cortex and the striatum. Our study indicated that attenuated spatiotemporal reactive astrogliosis, but not microglia activation, contributed to the neuroprotection of Ginseng pretreatment against ischemic damage.

Our *in vivo* research will not only allow us to clarify that Ginseng is able to rapidly elicit or boost endogenous cellular neuroprotection after stroke, but also provide new insight into the nature of Nrf2 in the context of ischemic stroke. This may open a window of opportunity to utilize these endogenous neuroprotection mechanisms in patients with stroke and other neurological diseases in clinic. To our knowledge, our study is the first to show evidence for the role of Nrf2 deficiency in long-term sensorimotor deficits and neuropathological outcome in pdMCAO-induced ischemic stroke in mice. The loss of Nrf2 exacerbated the acute sensorimotor deficits, the deterioration of ischemic damage and the progression of reactive astrogliosis, and delayed the long-term functional recovery from ischemic damage. These data further highlighted the vital role of Nrf2 in ischemic stroke pathogenesis.

In conclusion, the optimized method of the cylinder test depicts a more sensitive and reliable mean to detect sensorimotor deficits, which could be most instrumental for ischemic stroke research. Ginseng pretreatment protects against acute sensorimotor deficits and promotes its long-term recovery after pdMCAO, at least partly, through Nrf2 activation, highlighting the potential efficacy of oral consumption of Ginseng for stroke preventative intervention in patients who are at great risk of recurrent stroke or transient ischemic attack, that impedes the ischemic cascade and ultimately facilitates functional recovery. The attenuated spatiotemporal reactive astrogliosis contributes to the Nrf2 pathway related neuroprotection against acute ischemic outcome and substantially long-term sensorimotor deficits in the context of ischemic stroke under pdMCAO, providing the new insight into the cellular mechanism of Nrf2 in endogenous neuroprotection. Nrf2 deficiency led to deleterious progression following pdMCAO-induced ischemic stroke, enriching our acknowledgment of the natural properties of Nrf2 in health and neurological diseases. This *in vivo* work assessed the unique properties that Ginseng can provide the brain cells with resistance against acute and potentially chronic debilitating neurodegenerative conditions like ischemic stroke, and it highlighted the contribution of Nrf2 to Ginseng’ protective effect.

## Author Contributions

LL and SD: conceived the project and designed the experiments and prepared the manuscript with input from MKV. LL, MKV, VMF and YD: performed the experiments, data collection and analyzing. HK provided the Korean Red Ginseng. All authors commented and agreed with the final version of the manuscript.

## Conflict of Interest Statement

The authors declare that the research was conducted in the absence of any commercial or financial relationships that could be construed as a potential conflict of interest.
